# Design and Field Validation of an Offline Synchronized Multi-Sensor DAQ System for Bridge Structural Health Monitoring

**DOI:** 10.3390/s26134274

**Published:** 2026-07-05

**Authors:** Guillermo Alandí, Julia Irene Real, Salvador Mateo, Reynaldo Antonio Cabezas

**Affiliations:** 1 Institute for Multidisciplinary Mathematics, Polytechnic University of Valencia, Camino de Vera, s/n, 46022 Valencia, Spain; jureaher@tra.upv.es (J.I.R.); salmavil@upv.es (S.M.); 2 Idvia 2020 Horizonte 2020 SL, Plaza Bandas de Música 5, Local 11, 46013 Valencia, Spain; reynaldo.cabezas@idvia.es

**Keywords:** Structural Health Monitoring (SHM), Data Acquisition System (DAQ), offline synchronization, MEMS accelerometer, displacement sensors, Real-Time Clock (RTC)

## Abstract

Structural Health Monitoring (SHM) of large-span bridges requires dense sensor networks to accurately capture dynamic and kinematic behaviors. Traditional commercial systems rely on complex wiring or wireless protocols that frequently suffer from data loss, high power consumption, and synchronization phase errors, which are detrimental to Operational Modal Analysis (OMA). To address these limitations, this study presents the design, development, and field validation of a custom, offline-synchronized multi-sensor Data Acquisition (DAQ) system. Two specialized sensor nodes were developed: (i) an inertial node integrating a low-noise triaxial MEMS accelerometer (ADXL355); and (ii) a displacement node featuring a 24-bit Analog-to-Digital Converter (ADS1220) for displacement sensors. Both nodes share an ultra-low-power microcontroller (STM32L431) and utilize a local microSD storage strategy with an intermediate pseudo-SRAM buffer. To ensure precise temporal alignment without wireless communication overhead, each node incorporates a temperature-compensated Real-Time Clock (DS3231) for offline timestamp synchronization. The system was validated during a field campaign on the Spyckstraße bridge (Germany), deploying a hardware pool of 53 physical DAQ nodes to monitor 118 distinct geometric measurement points (106 inertial, 12 displacement) through a hybrid strategy of fixed and roving setups. The proposed system achieved reliable, low-noise measurements and enabled accurate extraction of operational mode shapes, demonstrating its viability as a robust, cost-effective solution for large-scale infrastructure monitoring.

## 1. Introduction

Structural Health Monitoring (SHM) has become an essential paradigm for assessing the performance, integrity, and remaining lifespan of civil infrastructures, particularly aging highway bridges [1]. Effective SHM systems rely on the continuous or periodic collection of structural data to identify damage before it compromises structural safety. Among the most relevant parameters to monitor are dynamic responses (accelerations), used to extract modal properties, and kinematic behaviors (displacements), used to evaluate the condition of bearings and expansion joints [2]. Consequently, modern SHM campaigns require high-density multi-sensor networks to accurately capture the global behavior of large-span structures [3,4].

Despite significant technological advancements, deploying high-density sensor networks on existing bridges remains a profound logistical and economic challenge. Traditional wired DAQ systems, such as those relying on Integrated Electronics Piezo-Electric (IEPE) sensors, offer high precision and perfect synchronization but are severely constrained by high installation costs, the logistical burden of heavy cabling, and susceptibility to electrical noise over long cable runs [5]. Conversely, Wireless Sensor Networks (WSNs) and IoT-based solutions have gained immense popularity due to their ease of installation and cable-free architecture [6,7]. WSNs often struggle with severe operational limitations in harsh civil environments, including packet loss, high power consumption due to continuous radio transmission, and complex time-synchronization challenges [8].

Recent studies on energy-efficient data transmission and signal synchronization [9,10,11] highlight that maintaining continuous phase-locked synchronization across distributed wireless nodes imposes a severe energy overhead. Conventional WSNs rely on active radio communications for timestamping, which rapidly depletes batteries and remains highly susceptible to packet loss. While some local-storage architectures exist to mitigate wireless transmission issues, they typically suffer from unpredictable SD-card write latencies that cause buffer overflows and data loss at high sampling rates.

Achieving precise time synchronization across dozens of distributed nodes is highly challenging due to clock drift and network latency [12]. In the context of Operational Modal Analysis (OMA), even microsecond-level time synchronization errors among sensor nodes can destroy the physical phase relationships between Degrees of Freedom (DOFs). For instance, Chen, Y. et al. [13] demonstrated that synchronization errors as small as 30 μs can result in noticeable errors during modal phase estimation, severely compromising the reliability of the modal identification process.

Recent developments in Micro-Electro-Mechanical Systems (MEMS) have provided a viable alternative, offering low-noise, low-power, and cost-effective inertial sensors that compete with traditional piezoelectric sensors for SHM applications [14]. Specifically, the ADXL355 MEMS accelerometer has been widely adopted in structural monitoring due to its ultra-low noise density and high resolution [15], and has been validated for experimental modal analysis in recent SHM applications, such as those conducted by Magdaleno, A. et al. [16]. However, most commercial MEMS-based WSN ecosystems are closed architectures that lack the flexibility to integrate external analog sensors, such as string potentiometers, which are essential for measuring relative displacements in bridge abutments. Furthermore, achieving reliable, high-precision synchronization across a dense network without relying on energy-intensive wireless gateways remains a significant technical gap.

To overcome these limitations, this paper proposes the design, development, and field validation of a custom, offline-synchronized DAQ system specifically tailored for high-density bridge SHM. The developed system diverges from traditional wireless approaches by utilizing a local-storage paradigm synchronized via high-precision Real-Time Clocks (RTC). The system comprises two specialized nodes sharing a unified ultra-low-power microcontroller (STM32L431): an inertial node featuring a high-resolution triaxial MEMS accelerometer (ADXL355), and a kinematic node equipped with a 24-bit Analog-to-Digital Converter (ADS1220) for linear displacement sensors. By stamping exact timestamps on the data and using an intermediate pseudo-SRAM buffer to facilitate burst-write storage to a local microSD card, the system achieves offline synchronization without the overhead and unreliability of wireless communications. The system’s performance was validated through a full-scale field campaign on the Spyckstraße mixed-girder bridge in Kleve, Germany, demonstrating its capability to support complex OMA and simultaneous multi-point displacement monitoring.

## 2. Materials and Methods

### 2.1. Preliminary Campaign

**Figure 1:**
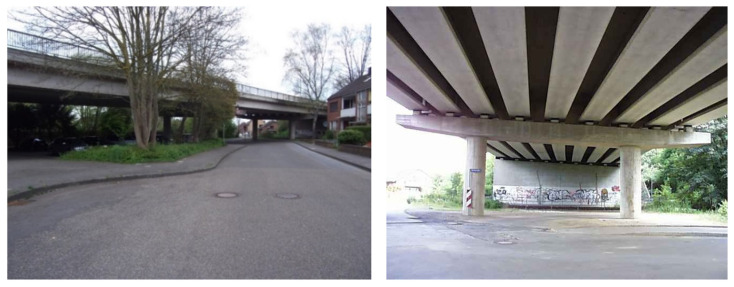
General view of the Spyckstraße bridge.

The design specifications for the proposed DAQ system were rigorously derived from a preliminary experimental campaign conducted on the Spyckstraße bridge, located in Kleve, Germany. Built in 1976, the structure is a 90.89 m long continuous mixed-girder bridge comprising three spans of 30.08 m, 30.73 m, and 30.08 m, as shown in Figure 1. The superstructure consists of two main steel plate girders working compositely with a reinforced concrete deck. Given its function as a vital urban artery carrying both regular vehicular traffic and heavy multi-axle freight, the bridge presented a complex dynamic environment. During this initial phase, a systematic evaluation of various measurement setups, ranging from high-end commercial systems to preliminary custom prototypes, was conducted. The objective was twofold: to characterize the structural dynamic response under operational conditions and to rigorously assess the physical and logistical constraints of instrumenting such a structure.

**Figure 2:**
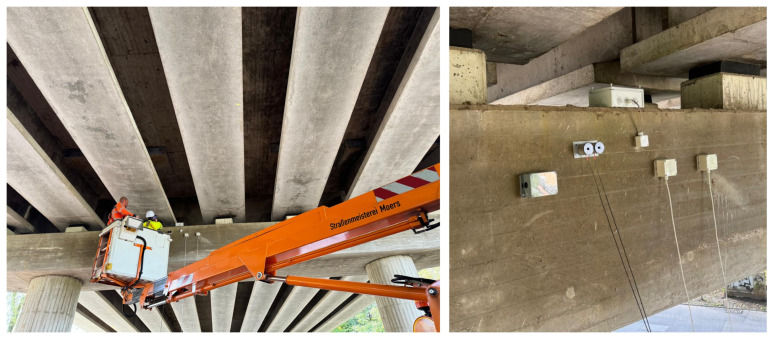
Installation of various types of sensors during the preliminary campaign.

To systematically evaluate the trade-offs between signal quality, logistical complexity, and system autonomy, four distinct DAQ architectures were deployed simultaneously (see Figure 2).

1. Commercial baseline: A high-precision wired system comprising IEPE accelerometers MMF-KB12VD (Metra Mess- und Frequenztechnik, Radebeul, Germany) connected to a 24-bit Dewesoft SIRIUS DualCore DAQ system (Dewesoft d.o.o., Trbovlje, Slovenia). This setup served as the gold standard for signal resolution. However, it required the sensors to be placed on the bridge deck while the DAQ and its controlling PC remained at the base, necessitating nearly a hundred meters of long cable runs and a connection to the main power grid.
2. Commercial MEMS evaluation: To evaluate alternative MEMS technologies against the IEPE baseline, a commercial evaluation board EVAL-ADXL355-PMDZ (Analog Devices Inc., Wilmington, MA, USA) was interfaced with a BeagleBone microcontroller (BeagleBoard.org Foundation, Dallas, TX, USA) for local data logging. While this proved the high sensitivity of the ADXL355 chip (Analog Devices Inc., Wilmington, MA, USA), the BeagleBone architecture still relied on external grid power and lacked the autonomy required for long-term campaigns.
3. Custom analog prototype: A wired analog system utilizing an ADXL354BEZ-RL sensor (Analog Devices Inc., Wilmington, MA, USA) with 4–20 mA current loops connected to a BeagleBone-based DAQ with an ADS8688AIDBT ADC (Texas Instruments, Dallas, TX, USA). This 4–20 mA architecture is the robust, proprietary solution employed by the authors for continuous, multi-year permanent bridge monitoring campaigns. It was tested to see if its inherent noise immunity over long cables could mitigate the issues seen in standard analog setups.
4. Custom Digital Prototype (Wireless/Autonomous): A fully integrated digital node based on the IIS3DWB sensor (STMicroelectronics, Geneva, Switzerland) and an STM32L431CCT6TR microcontroller (STMicroelectronics, Geneva, Switzerland) powered by D-cell batteries, designed specifically for rapid, cable-free deployment.

**Figure 3:**
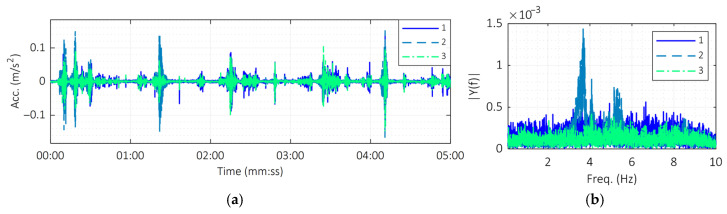
Data recorded with custom analog prototype longitudinal (1), vertical (2) and transverse (3) channels: (**a**) Accelerogram in the center-span section of the north span; (**b**) Frequency content of the recording.

The preliminary data from these setups revealed that the ambient vibration amplitudes were predominantly in the micro-g range. The comparison demonstrated that while the high-end Dewesoft setup provided excellent resolution, routing extensive coaxial cables proved operationally inefficient, costly, and susceptible to logistical hazards on an active bridge. The custom 4–20 mA analog system (see the graphs in Figure 3a,b), while extremely robust and highly suitable for long-term permanent installations, retained the severe cabling burden and reliance on external grid power, making it logistically impractical for high-density, temporary OMA campaigns.

Conversely, the battery-powered STM32-IIS3DWB digital prototype highlighted the logistical superiority of autonomous, cable-free local storage. However, spectral analysis revealed that the noise floor of the IIS3DWB proved insufficient to resolve the extremely low-amplitude ambient vibrations of the Spyckstraße bridge’s stiff composite deck.

The structural assessment also required the simultaneous monitoring of microscopic relative displacements at the elastomeric bearings and expansion joints, highlighting the need to integrate high-resolution kinematic sensors into the campaign without reintroducing cables.

Based on these empirical findings, the core minimum technical requirements for the new hardware were strictly established to ensure data acquisition and subsequent OMA processing. These specifications are summarized in Table 1.

### 2.2. Hardware Architecture of the DAQ Nodes

To fulfill the requirements established during the preliminary campaign, modular, dual-node hardware architecture was engineered. Instead of integrating all sensors into a single, bulky unit that would compromise placement flexibility, two specialized Printed Circuit Boards (PCBs) were developed: Node 1, dedicated to inertial measurements, and Node 2, dedicated to displacement measurements.

**Figure 4:**
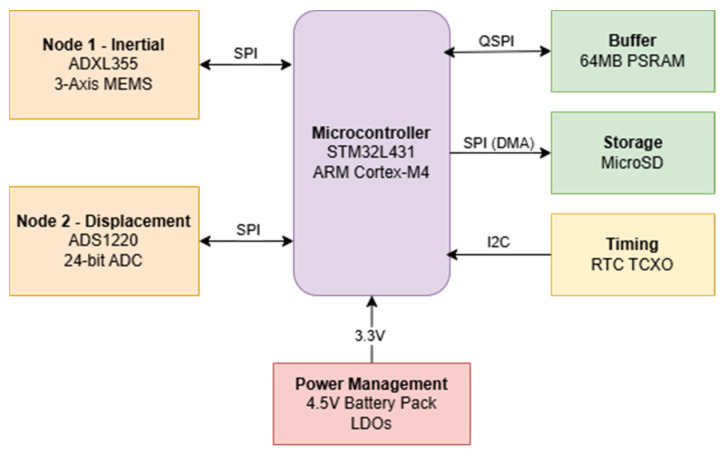
Hardware architecture flow chart.

Both nodes share an identical central processing, power management, and local storage architecture. This design philosophy not only streamlines firmware development but ensures identical temporal behavior and latency across the entire network, a prerequisite for offline synchronization. Figure 4 shows the architecture flowchart.

**Figure 5:**
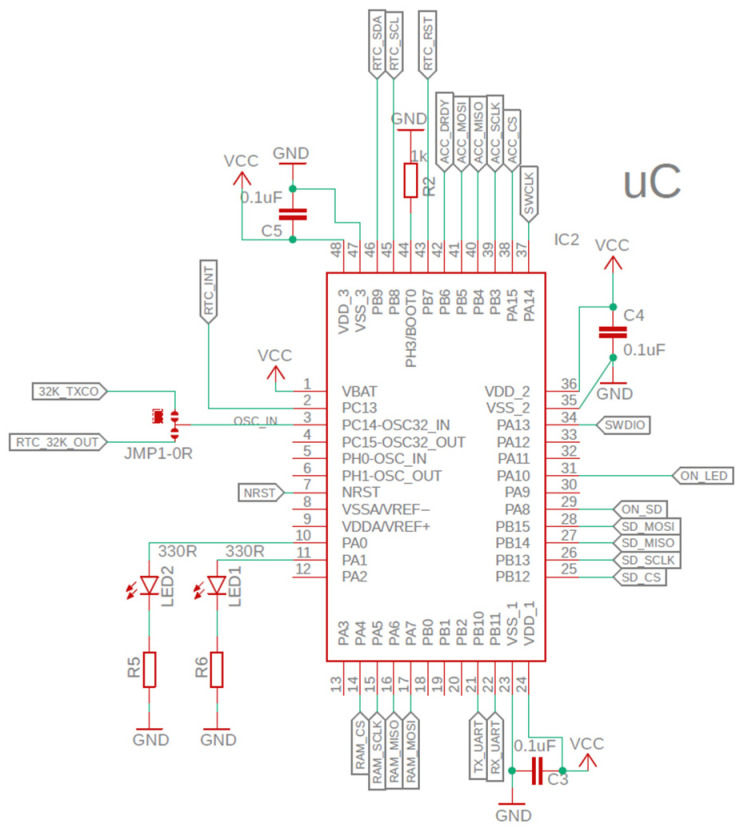
MCU STM32L41 circuit diagram.

The core computational and control unit of both nodes is the STM32L431CCT6, an advanced 32-bit ARM Cortex-M4 microcontroller (Arm Ltd., Cambridge, UK) equipped with a Floating-Point Unit (FPU) [17]. This specific MCU was selected for its ultra-low-power capabilities tailored for autonomous IoT applications. Operating at a core frequency of up to 80 MHz, it features scalable power modes and a dynamic current consumption of approximately 100 µA/MHz. The hardware architecture relies heavily on the MCU’s multi-channel Direct Memory Access (DMA) controllers. By utilizing DMA, sensor data is continuously streamed from the SPI peripherals directly into the MCU’s SRAM without requiring CPU intervention. The suitability of the STM32L4 family for such demanding tasks has been corroborated by recent advancements in autonomous SHM nodes, as detailed by Guo et al. [18], demonstrating its optimal balance between executing high-speed, non-blocking data transfers and maintaining minimal energy consumption through aggressive sleep modes between sampling events. The schematic diagram of the MCU integration is presented in Figure 5.

A challenge in standalone DAQ systems is the non-volatile storage bottleneck. Standard microSD cards exhibit high peak current consumption (often exceeding 100 mA during active write cycles) and highly non-deterministic write latencies. These unpredictable latencies, sometimes reaching up to 250 ms during internal flash page erasures and wear-leveling cycles managed by the card’s internal controller [19], can severely disrupt continuous high-frequency sampling. As highlighted in recent low-power DAQ optimization studies by Khan et al. [20], streaming continuous sensor data directly to an SD card over a standard SPI bus often temporarily blocks the MCU, leading to catastrophic buffer overflows, data loss, and timing jitter.

**Figure 6:**
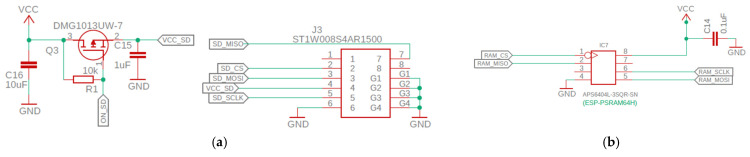
Memory circuit diagrams: (**a**) MOSFET and microSD; (**b**) PSRAM.

To completely mitigate this bottleneck, an external 64 MB Pseudo-SRAM was integrated via a Quad-SPI (QSPI) interface [21]. The PSRAM acts as an ultra-fast, predictable, and low-power intermediate circular buffer (see Figure 6b). During operation, the MCU continuously offloads high-resolution sensor data into the PSRAM. Meanwhile, the energy-intensive microSD card is kept completely powered off via a dedicated MOSFET load switch (see Figure 6a). Only when the PSRAM buffer reaches a predefined high-water mark does the MCU quickly power up the SD card, perform a massive DMA-driven burst-write, and immediately power the SD card down again. This architecture guarantees zero data loss and significantly extends battery life.

**Figure 7:**
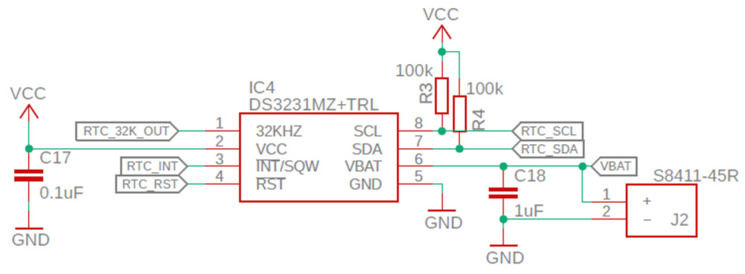
RTC circuit diagram.

For offline temporal synchronization, instead of relying on energy-intensive wireless synchronization protocols, which are highly susceptible to packet loss and variable latencies in dense civil environments, each node operates strictly autonomously. Each board incorporates a DS3231MZ RTC communicating via the I2C bus [22]. The DS3231MZ (see Figure 7) features an internal Temperature-Compensated Crystal Oscillator (TCXO) that guarantees a strict accuracy of ±5 parts per million (ppm) across the entire industrial temperature range (−40 °C to +85 °C).

DS3231MZ provides a highly stable 1 Hz square wave output. In this hardware design, the PPS signal is hardwired directly to an external interrupt (EXTI) pin on the STM32. This hardware interrupt allows the MCU to precisely align its internal high-speed timers to the absolute RTC time every second, eliminating software polling delays. The efficacy of utilizing such high-precision RTCs and PPS-driven offline synchronization strategies in structural monitoring has been recently validated by Nilnoree et al. [23], demonstrating their capability to maintain strict phase coherence across isolated sensor networks over multi-week deployments without the severe energy penalties of wireless communication. To ensure clarity and reproducibility, the primary electronic components composing both DAQ nodes are summarized in Table 2.

### 2.3. Digital Accelerometry Module: Node 1

Node 1 is designed to capture the dynamic response of the bridge. The core sensing element of this module is the ADXL355, a highly integrated, low-noise, triaxial MEMS accelerometer [15]. Based on the findings of the preliminary campaign, which highlighted the micro-g nature of the bridge’s ambient vibrations, the ADXL355 was selected primarily for its industry-leading noise density profile of just 25 µg/√Hz across all three axes, paired with exceptionally low thermal drift (0.15 mg/°C) [15].

To contextualize this component selection and justify its suitability for OMA, Table 3 presents a comprehensive performance benchmark comparing the ADXL355 against other high-end industrial MEMS accelerometers commonly evaluated for SHM applications: the SCA3300-D01 (Murata Manufacturing Co., Kyoto, Japan) and the IIS3DWB.

As detailed in Table 3, while the IIS3DWB focuses on high-frequency vibration analysis (with a bandwidth up to 6.3 kHz), its higher noise floor (45 $\mu g/\sqrt{\mathrm{Hz}}$) and temperature drift (0.3 mg/°C) obscure the micro-g ambient responses of stiff composite bridge decks. The Murata SCA3300-D01 offers noise characteristics (37 $\mu g/\sqrt{\mathrm{Hz}}$) suitable for civil engineering; however, its active current consumption (1.2 mA) is six times higher than the ADXL355, and its bandwidth is limited to 70 Hz. For an autonomous, battery-operated sensor network intended for multi-week deployments, the ADXL355 provides the optimal balance. It achieves the lowest noise floor (25 $\mu g/\sqrt{\mathrm{Hz}}$) and superior ADC resolution (20-bit versus 16-bit) to resolve microscopic structural dynamics, while maintaining a low power profile (200 µA) to maximize the system’s operational autonomy.

Recent literature has extensively validated the performance of the ADXL355 against traditional piezoelectric sensors. As previously noted by Magdaleno et al. [13], it has demonstrated its capacity to accurately extract high-fidelity acceleration response functions for Experimental Modal Analysis. Its reliability has been recently proven in experimental shake table laboratory settings for SHM, as detailed by Occhipinti et al. [24].

Unlike its analog counterpart (the ADXL354) tested during the preliminary phase, the ADXL355 incorporates a built-in 20-bit ADC alongside digital low-pass and high-pass filtering capabilities. Processing the analog signal internally within the shielded MEMS package provides two major advantages for the node’s architecture: (i) it eliminates the need for external signal conditioning circuitry on the PCB, drastically reducing the physical footprint and the susceptibility to electromagnetic interference (EMI); and (ii) it offloads anti-aliasing filtering tasks from the main STM32L431 microcontroller.

**Figure 8:**
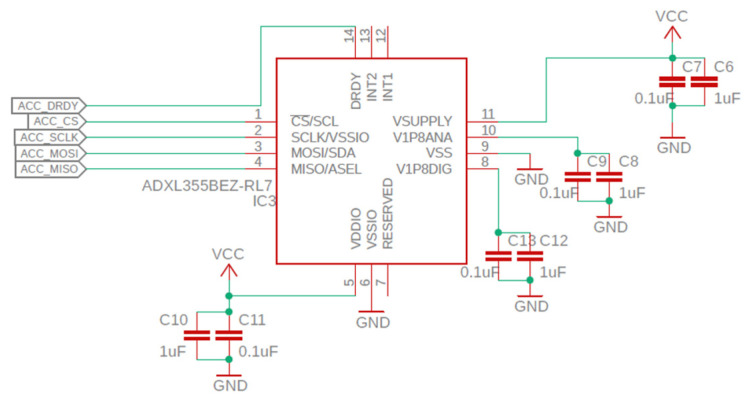
ADXL355 MEMS sensor circuit diagram.

The integration between the ADXL355 and the MCU is achieved via a dedicated, high-speed SPI bus interface. Rather than triggering a costly hardware interrupt for every single sample (which at 1000 Hz would severely burden the MCU with context-switching overhead), the firmware leverages the ADXL355’s internal 96-level FIFO buffer. The sensor continuously samples the structural vibrations driven by its own highly stable internal clock at a strict Output Data Rate (ODR) of 1000 Hz, storing the 20-bit data internally. As detailed in Section 2.5, the MCU actively polls the FIFO status and reads the data in discrete multi-sample blocks, significantly optimizing the SPI bus utilization and minimizing timing jitter. Figure 8 shows the circuit diagram.

**Figure 9:**
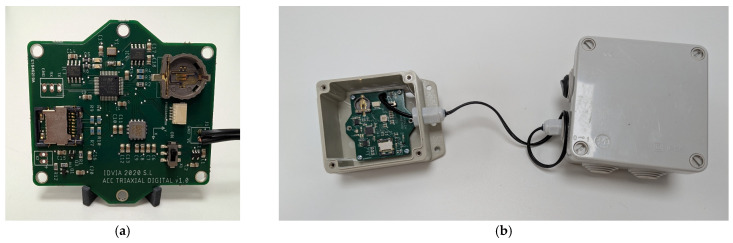
Digital accelerometry module: (**a)** PCB manufactured; (**b**) Complete node with enclosure and batteries.

From a mechanical and structural perspective, capturing low-amplitude, low-frequency vibrations imposes strict constraints on the PCB layout (see Figure 9a) and on the node’s physical packaging. A design criterion was the physical decoupling of the power source from the sensitive inertial measurement unit. To achieve this, the PCB containing the ADXL355 was mounted inside a compact, lightweight plastic enclosure, which was kept entirely independent from the heavy alkaline battery pack. The batteries were housed in a separate enclosure and connected to the sensor node via a short, flexible power cable, as shown in Figure 9b.

As established in fundamental experimental modal analysis theory [25], attaching a substantial lumped mass directly to the measurement point introduces a mass loading effect. This phenomenon alters the local dynamic stiffness of the structure, artificially lowering its natural frequencies and distorting modal amplitudes. Integrating heavy batteries within the same housing as the accelerometer increases the risk of introducing low-frequency parasitic resonances due to the mechanical flexibility of a heavier enclosure or microscopic internal movements of the batteries themselves, a concern previously highlighted in the literature regarding WSN deployments [3].

By physically isolating the sensor’s mass, the proposed design ensures a stiff, direct coupling path from the bridge structure to the MEMS chip. The MEMS component was placed at the center of the PCB, directly adjacent to rigid mechanical mounting holes. This topological decision moves the fundamental resonant frequency of the node well beyond the structural bandwidth of interest, thus fully preventing phase distortion and mechanical amplification errors during the OMA.

### 2.4. Displacement Module: Node 2

While Node 1 focuses on the global dynamic response of the bridge, a complete structural assessment also demands looking at local kinematic behavior. Tracking relative linear displacements at expansion joints and elastomeric bearings provides direct insight into thermal expansion cycles, traffic-induced micro-movements, and early signs of bearing lock-up [2]. Because capturing these slow, structural shifts requires a completely different sensing approach than measuring vibrations, Node 2 was developed as a dedicated kinematic module.

At the core of Node 2 sits an ADS1220, an ultra-low-power, 24-bit ADC [26]. This specific ADC was chosen because kinematic monitoring involves low-frequency signals but demands extremely high resolution. Given that the TE Connectivity SP1-12-3 potentiometer has a full stroke of 317 mm, capturing sub-millimeter structural shifts translates into microscopic voltage changes. The 24-bit resolution ensures that these subtle variations are not lost in quantization noise. To justify the selection of the ADS1220, Table 4 presents a technical comparison with two alternatives, namely, the internal ADC of the STM32L431 microcontroller, and the AD7124-4, another high-end 24-bit Sigma-Delta converter common in industrial instrumentation.

The rationale for utilizing an external 24-bit ADC over the built-in microcontroller ADC becomes evident through this comparison. Given that the SP1-12-3 potentiometer has a full stroke of 12.5 inches (317.5 mm), a 12-bit SAR ADC yields a theoretical maximum resolution of approximately 0.077 mm per step. In practice, due to electrical noise, the effective resolution would be even coarser, obscuring critical traffic-induced micro-movements. The internal ADC lacks a true Programmable Gain Amplifier (PGA) and hardware-level 50/60 Hz filtering, making it highly susceptible to electromagnetic interference from bridge lighting and power lines.

While the AD7124-4 provides comparable 24-bit precision and integrated filtering, its higher active current consumption (255 µA) and excessive sampling speed (19.2 kSPS) are unnecessary for tracking slow structural displacements. The ADS1220 provides the optimal balance. Its duty-cycle mode lowers power consumption to 120 µA, preserving battery life. The 24-bit resolution ensures that subtle structural variations are recorded with sub-micron theoretical precision, keeping the quantization noise well below the mechanical noise floor of the bearing itself.

**Figure 10:**
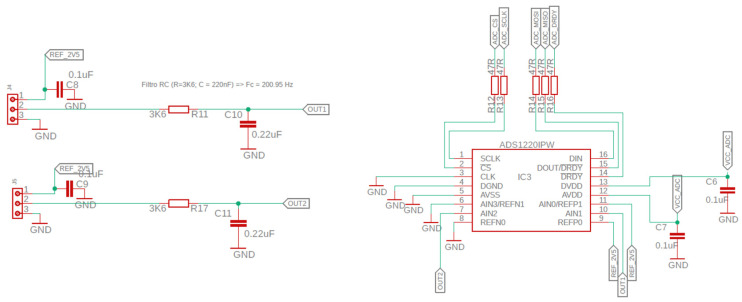
ADS1220 circuit diagram.

The highly integrated analog front-end of the ADS1220 further simplifies the board design (see Figure 10). With its built-in low-noise Programmable Gain Amplifier (PGA) handling gains up to 128 V/V, the resistive string potentiometers can be connected directly to the ADC. This design choice bypasses the need for external amplification stages, which often introduce unwanted electrical noise on the PCB. In an active bridge environment, this inherent rejection is invaluable for filtering out electromagnetic interference (EMI) from overhead power lines and street lighting [26].

**Figure 11:**
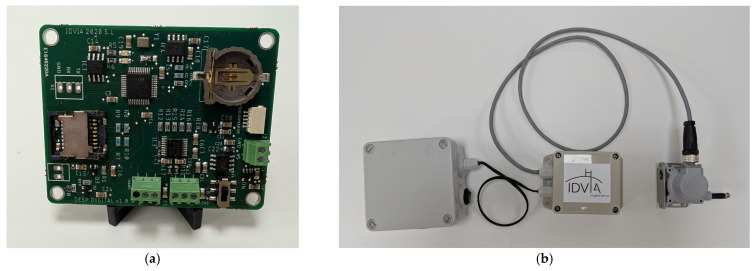
Displacement module: (**a**) PCB manufactured; (**b**) Complete node with enclosure, batteries and TE Connectivity SP1-12-3 analog sensor.

For the potentiometers to yield stable readings, their excitation voltage must be extremely clean. Therefore, Node 2 uses a dedicated Low-Dropout Regulator (LDO) to supply a tightly regulated 3.3 V directly to the external sensor. The analog signal returning from the potentiometer’s internal voltage divider then passes through a passive RC anti-aliasing filter placed as physically close to the ADC input pins as possible before entering the differential inputs. Figure 11a shows the resulting PCB, as well as the battery housing assembly and the connected potentiometer in Figure 11b.

To keep the entire sensor network temporally aligned, the firmware driving Node 2 utilizes a hardware interrupt strategy. The ADS1220 operates in continuous conversion mode over the SPI bus. Upon completing a 24-bit sample, the ADC pulls its Data Ready (DRDY) pin low. This triggers an external hardware interrupt (EXTI) flag on the STM32L431. The MCU services this flag, retrieves the displacement data, and appends a high-resolution absolute timestamp alongside a sequential hardware counter. This data block is then swiftly pushed into the PSRAM buffer. By timestamping each individual kinematic sample, the displacement data can be seamlessly merged with the acceleration matrices during offline post-processing.

### 2.5. Firmware Architecture

The firmware driving the STM32L431 microcontroller was developed in C, utilizing hardware abstraction layers (HAL) to manage peripherals. Addressing the concerns often associated with low-power DAQ design, the firmware avoids a complex, per-sample interrupt-driven architecture. Instead, it implements a highly robust, sequential state machine divided into three distinct phases: Acquisition, Transfer, and Sleep. This FIFO-polling strategy guarantees zero data loss at high sampling rates (1000 Hz) while maintaining strict temporal control.

**Figure 12:**
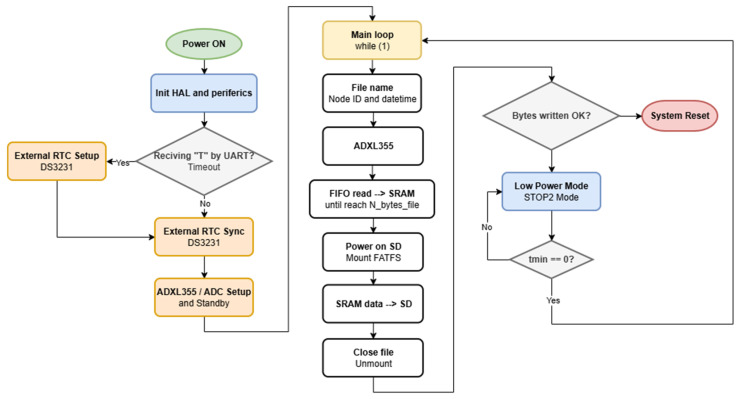
Firmware architecture flowchart of inertial node.

As illustrated in the flowchart in Figure 12, at startup, the MCU initializes all peripherals (GPIO, I2C, SPI, UART, and FATFS) and determines its operational configuration (e.g., node ID, sampling duration, and sleep interval tmin). Before autonomous deployment, strict temporal synchronization is enforced. The node illuminates its status LEDs and transmits a readiness byte (‘T’) over the USART3 serial interface. It then waits to receive a strictly formatted 9-byte time payload from a master computer via a custom Python script (version 3.10, Python Software Foundation, Wilmington, DE, USA). This payload configures the external DS3231 RTC, which subsequently disciplines STM32 internal RTC. Once synchronized, the MCU powers down the sensors and enters its primary infinite loop.

Phase A: Sensor to SRAM. The active cycle begins by generating a unique timestamped filename (e.g., NodeID_YY-MM-DD_HHmmss.txt) derived from the synchronized internal RTC. To accommodate the distinct sensor architectures, Phase A executes specific routines based on the defined node type:

- Node 1. The MCU powers on the ADXL355 sensor and enters a continuous loop to capture data at 1000 Hz, the Output Data Rate (ODR) driven by the sensor’s internal oscillator. The MCU actively polls the sensor’s FIFO buffer; once sufficient data is ready, it reads a 63-byte payload via the SPI bus, which represents seven complete triaxial acceleration samples. To preserve data integrity during post-processing, the firmware appends a 1-byte hardware counter, forming a 64-byte block. This block is written on the external SRAM buffer. By routing the continuous, high-frequency data stream directly to the fast SRAM instead of the microSD card, the system entirely avoids the unpredictable write latencies that typically cause buffer overflows in conventional loggers.
- Node 2: Given the lower sample rates required for structural displacements, this module relies on an interrupt-driven approach. The ADS1220 signals a completed conversion via its DRDY pin, raising an EXTI flag. The MCU detects this flag, reads the 24-bit data, and constructs a tightly packed 12-byte payload. This block comprises a high-resolution absolute timestamp, the 32-bit cast displacement value, a hardware counter, and a delimiter. This 12-byte block is subsequently pushed to the PSRAM.

Phase B: SRAM to SD. Upon reaching the predefined memory threshold for the measurement window, the MCU deactivates the sensor and transitions to the storage phase. Power is supplied to the microSD card via a dedicated GPIO-controlled MOSFET, and the FAT32 file system is mounted. The firmware then efficiently reads large 512-byte blocks from the SRAM and writes them directly to the SD card in a continuous, high-speed burst. The system performs a byte-count verification to prevent data corruption. If a write error or mismatch is detected, a hardware failsafe triggers an automatic system reset to recover the node. Following a successful transfer, the file is closed, the file system is unmounted, and the SD card is physically powered off to eliminate quiescent current draw.

Phase C: Low-Power Sleep. To maximize battery autonomy, the node enters a sleep phase between measurement intervals. Before sleeping, the MCU queries the external DS3231 TCXO to correct any minor drift accumulated by the internal RTC during the active phases. The system ticks are then suspended, and the MCU enters a low-power state. A periodic wake-up routine acts as a heartbeat and evaluates the current RTC time against the predefined sleep interval. The MCU remains in this sleep state until the current minute perfectly aligns with the target multiple. Once aligned, the MCU fully awakens, and the operational cycle restarts, maintaining temporal synchronization with the rest of the network.

### 2.6. Field Validation

**Figure 13:**
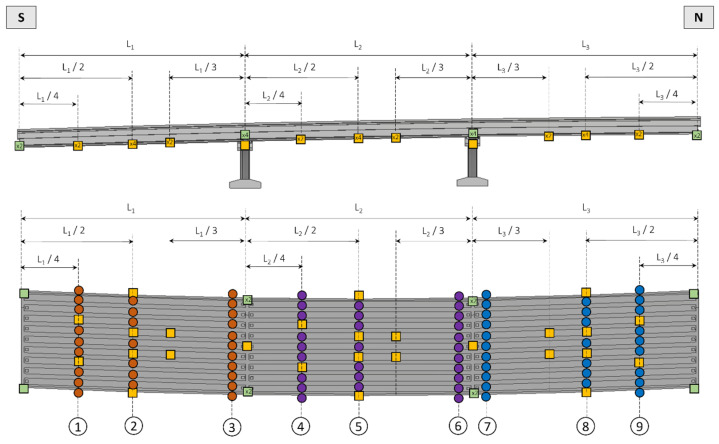
Node installation diagram on Spyckstraße bridge.

To rigorously evaluate the performance of the proposed DAQ architecture under real-world operational conditions, a full-scale field validation campaign was conducted on the Spyckstraße bridge. The deployment consisted of 53 independent sensor nodes in total, to cover 118 measurement points as shown in Figure 13: 26 fixed inertial modules (Node 1), 15 mobile inertial modules (Node 1) and 12 displacement modules (Node 2). Managing a network of this scale on an active highway bridge without the logistical burden of running hundreds of meters of cables immediately demonstrated the operational advantage of the autonomous, local-storage design.

To comprehensively capture the dynamic behavior of the bridge using the inertial nodes, a hybrid strategy combining fixed and mobile measurements was implemented. To achieve high-density spatial resolution without incurring the prohibitive cost of manufacturing 106 individual inertial nodes, the remaining 15 inertial modules were used as roving sensors. These mobile units were systematically moved across densely mapped measurement grids to cover 80 additional points along the longitudinal girders and the concrete deck. For each configuration setup, the 15 roving nodes were installed, left to measure ambient vibrations for 2 to 4 h, and then uninstalled and relocated to the next set of grid positions. Through this multi-setup strategy, combined with the continuous data from the 26 reference nodes, the 53 physical DAQ nodes successfully measured a total of 118 distinct geometric points (106 inertial locations and 12 bearing displacements) across the bridge. Inertial nodes were permanently installed at optimal predefined locations, specifically at the mid-span (L/2), one-third span (L/3), and one-quarter span (L/4) sections of the main girders. This multi-setup strategy, combined with the continuous data from the reference nodes, allowed for the subsequent merging of mode shapes during the OMA [27].

**Figure 14:**
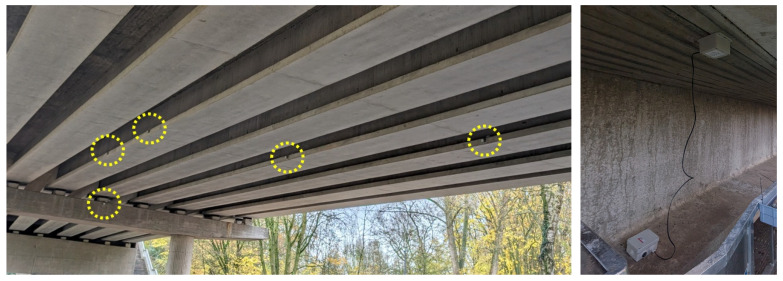
Inertial nodes installation on Spyckstraße bridge. Yellow circles indicate the placement of some nodes.

Ensuring a rigid mechanical connection between the sensor and the structure is critical for high-fidelity vibration measurements. The compact enclosures housing the inertial PCBs were firmly attached directly to the steel girders and concrete deck (see Figure 14). This was achieved by drilling shallow holes (not exceeding 3 cm in depth) into the concrete and using screws and plastic wall plugs. This method provided the necessary rigidity without compromising the structural integrity. Upon completion of the campaign and the removal of the sensors, these shallow holes were carefully filled and sealed with quick-setting cement to ensure the internal steel reinforcement remained fully protected from environmental exposure. As detailed in Section 2.3, the battery packs were housed separately and secured nearby, completely preventing any mass-loading effects or low-frequency enclosure rattling from corrupting the micro-g acceleration data.

**Figure 15:**
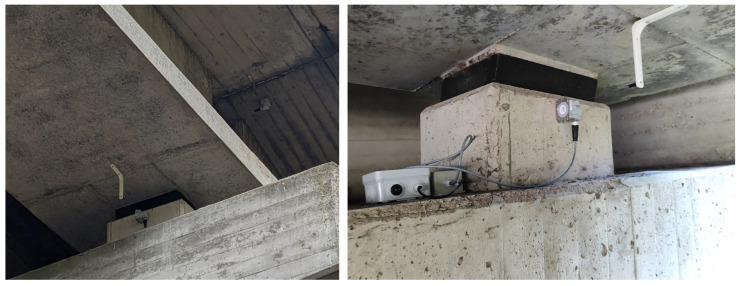
Displacement nodes installation on Spyckstraße bridge.

Simultaneously, the 12 kinematic modules (Node 2) were deployed to monitor the elastomeric bearings, as shown in Figure 15. The TE Connectivity SP1-12-3 string potentiometers were positioned to capture the relative movements between the substructure and the superstructure. At the abutments, the potentiometers were installed longitudinally, anchored between the abutment face and the outermost girders. At the intermediate piers, a more comprehensive measurement strategy was adopted: for each side of the pier, two potentiometers were installed between the pier and the girders. One oriented longitudinally and the other transversely, accounting for 4 dual-axis measurement locations.

Prior to the physical deployment on the bridge, it was imperative to establish a common time baseline across the network. After inserting the dedicated coin-cell backup batteries for the DS3231 RTCs, each node was individually connected to a master computer via a high-speed serial interface (115,200 baud). A custom Python script was executed to read the host computer’s precise local time and transmit a strictly formatted binary payload directly to the microcontroller. Upon receiving this custom command frame, the MCU programmed its local RTC. This initialization procedure guaranteed that all 65 nodes shared an identical, synchronized temporal starting point before commencing data capture.

Once deployed on the Spyckstraße bridge, the nodes operated completely autonomously, saving their raw 64-byte binary data packets directly to their respective microSD cards. To guarantee full traceability and to facilitate the automated parsing of thousands of generated files, the firmware automatically assigns a unique identifier to every single generated .txt file. After the campaign concluded, the SD cards were retrieved, and these files were processed through custom MATLAB scripts (version R2023a, MathWorks, Natick, MA, USA).

## 3. Results

### 3.1. System Performance and Data Integrity

Upon concluding the multi-week measurement campaign on the Spyckstraße bridge, all 38 nodes were retrieved alongside their respective microSD cards. Despite the harsh environmental conditions and the continuous vibrations from heavy freight traffic, none of the nodes experienced power failures or structural detachment. The Type-C alkaline battery packs maintained a stable voltage profile throughout the deployment, validating the ultra-low-power, interrupt-driven firmware design discussed in Section 2.5.

To substantiate this operational stability and address the power consumption constraints typical of standalone loggers, a quantitative energy profile of the nodes was established. Each node is powered by three Type-C alkaline batteries connected in series, providing a nominal 4.5 V to the primary LDO and offering a total capacity of approximately 7800 mAh. Based on the selected hardware components and the firmware operation cycle, the current draw varies across the three operational phases.

During Phase A, the MCU remains in active run mode to poll the ADXL355 FIFO and transfer data to the PSRAM. The combined active current of the MCU, the MEMS sensor, and the PSRAM write cycles yields a total consumption of approximately 18.3 mA. In Phase B, the dedicated MOSFET activates the microSD card, producing a transient current spike of up to 80 mA. However, because the data is pre-buffered in the fast PSRAM, this high-energy phase lasts less than 2 s for every 15 min of recorded data. Finally, during Phase C, the MCU enters STOP2 mode, and all peripherals are placed in standby, reducing the entire board’s consumption to under 200 µA. For standard periodic SHM campaigns recording 15 min per hour, the time-averaged current drops to roughly 4.81 mA, extending the theoretical battery life to over 67 days.

Data extraction and parsing were streamlined by the sequential file naming convention. Evaluating the 1-byte hardware counter embedded within each 64-byte payload confirmed zero dropped frames or buffer overflows across the entire campaign. This data continuity demonstrates that treating the PSRAM as the primary circular buffer and restricting the SD card to brief, high-speed burst writes circumvents the latency bottlenecks typical of conventional local-storage loggers.

**Figure 16:**
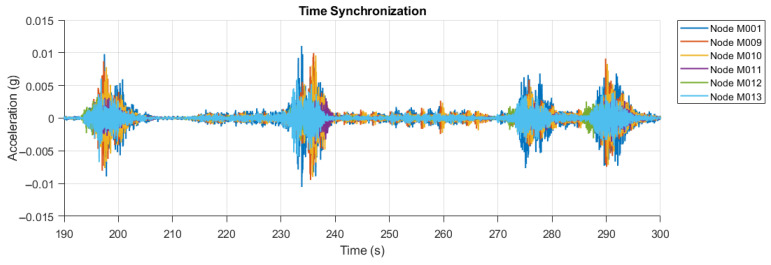
Magnified time-history window demonstrating time synchronization across multiple independent nodes during a traffic event.

The offline synchronization strategy was performed as expected. Cross-correlation analysis between stationary reference nodes demonstrated that the phase relationships remained locked over the entire multi-day period, achieving a temporal alignment accuracy. To visually illustrate this temporal precision, Figure 16 presents a magnified time-history window of the vertical acceleration recorded simultaneously by independent reference nodes located at the equivalent south-span cross-section.

### 3.2. Signal Quality, Spectral Analysis, and SNR

**Figure 17:**
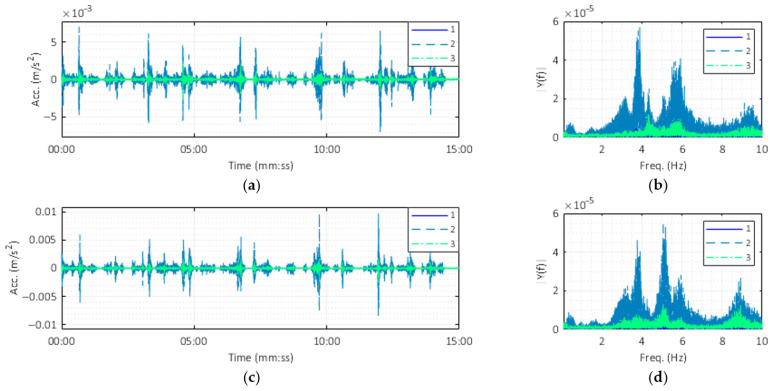
Data processed with longitudinal (1), vertical (2) and transverse (3) channels: (**a**) Accelerogram in the center-span section of the north span; (**b**) Frequency content of the recording; (**c**) Accelerogram in the center-span section of the center span; (**d**) Frequency content of the recording.

The primary objective of the field validation was to quantitatively verify the DAQ system’s capacity to resolve micro-g ambient vibrations without the signal being buried in the electronic noise floor. To evaluate this, synchronized 15-min acceleration records, sampled at a high-speed rate of 1000 Hz, were analyzed using custom MATLAB routines as shown in Figure 17.

The time-domain data showed clean, zero-mean dynamic responses. For instance, simultaneous recordings from two different nodes along the deck yielded vertical acceleration Root Mean Square (RMS) values of 1.031 × 10^−3^ m/s^2^ and 8.65 × 10^−4^ m/s^2^, respectively. Converting these time-domain signals into the frequency domain via Fast Fourier Transform (FFT) provided a direct assessment of the sensor’s spectral performance. The analysis of the concurrent datasets revealed sharply defined structural resonant peaks at 3.94 Hz and 3.77 Hz.

Despite these microscopic excitation levels, the estimated Signal-to-Noise Ratio (SNR) for these modes was calculated at 25.10 dB and 27.50 dB, respectively. An SNR exceeding 25 dB in an ambient vibration context is exceptional, indicating that the power of the structural signal is over 300 times greater than the background noise floor. This empirically validates the selection of the ADXL355 sensor and analog/digital physical isolation implemented on custom PCB.

While a comprehensive OMA and detailed structural health assessment of the Spyckstraße bridge are beyond the scope of this hardware-focused paper and will be presented in a separate companion study, preliminary processing using standard Frequency Domain Decomposition (FDD) [28] confirmed the integrity of the data. The high SNR allowed the algorithms to identify the fundamental bending and torsional mode shapes, confirming that the proposed autonomous architecture delivers data ready for advanced digital twin updating.

**Figure 18:**
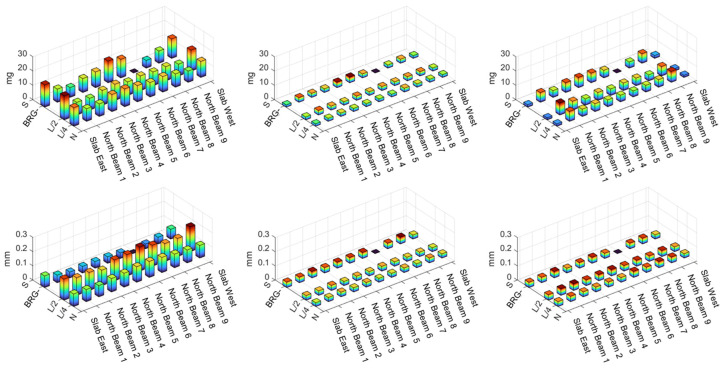
Median of the maximum acceleration (**top**) and displacement (**bottom**) values recorded with the mobile sensor scheme located in the north span. Vertical axis (**left**); longitudinal axis (**center**); transverse axis (**right**).

Beyond purely spectral analysis, the roving sensor scheme permitted a detailed evaluation of maximum local responses under operational traffic. Figure 18 illustrates the median of the maximum acceleration and displacement values recorded during the mobile setups located in the north span.

The ability to simultaneously capture these low-amplitude transient peak accelerations and their corresponding integrated displacements across all three principal axes (vertical, longitudinal, and transverse) further confirms the high dynamic range and low-frequency stability of the inertial nodes. This transient local data is crucial for validating localized stiffness degradation before integrating the responses into the global Operational Modal Analysis.

### 3.3. Operational Modal Analysis

To validate the DAQ system’s suitability for structural identification, an Operational Modal Analysis (OMA) was conducted using the synchronized data collected across the 118 geometric measurement points. The high-density spatial grid, achieved through the hybrid fixed and roving measurement strategy, provided the necessary Degrees of Freedom (DOFs) to accurately map the bridge’s global dynamic behavior.

**Figure 19:**
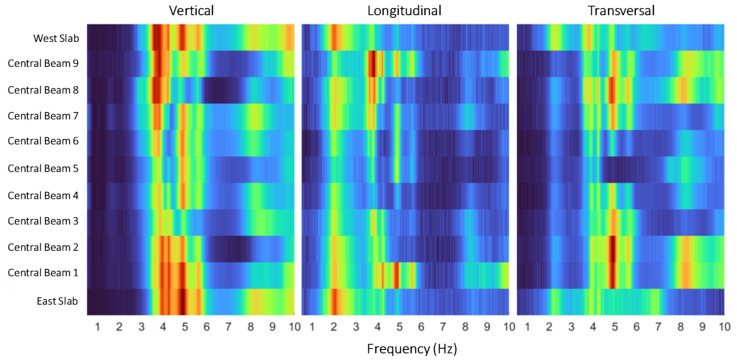
PSD obtained in the structural elements of the central span. Vertical axis (**left**); longitudinal axis (**center**); transverse axis (**right**).

The Frequency Domain Decomposition (FDD) [28] algorithm was applied to the acceleration time-histories. The high SNR of the ADXL355 nodes allowed for the identification of the structural poles without the need for excessive data filtering. To identify the resonant frequencies, the Power Spectral Density (PSD) was evaluated across the different measurement axes (see Figure 19). The detailed extraction of these modal parameters and their application in numerical model calibration are extensively discussed in a companion study by the authors [29].

The identified experimental frequencies were subsequently used as the baseline for the structural assessment. Table 5 summarizes the natural frequencies resulting from this process, which directly fed the calibration and validation of the structure’s numerical model.

**Figure 20:**
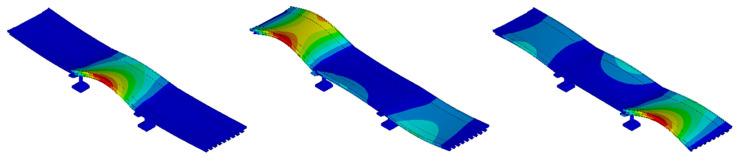
Deformed shapes associated with the vertical bending modes of each span in the Digital Twin of the Spyckstraße Bridge.

The experimental mode shapes corresponding to these frequencies were extracted and matched against the numerical model to verify the structural integrity. Figure 20 illustrates the deformed shapes for the primary structural responses.

The smooth, well-defined contours of these mode shapes empirically validate the robustness of the offline RTC-based synchronization architecture. In OMA, even microsecond-level synchronization drifts between distributed nodes result in modal phase errors, which visually distort the extracted mode shapes. The clean geometrical deformation patterns captured by the proposed DAQ network confirm that the sub-millisecond temporal alignment between the independent hardware units ensures that the physical phase relationships of the structure are preserved, delivering high-fidelity modal data suited for FEM updating and Digital Twin calibration [25].

### 3.4. Kinematic Bearing Behavior

**Figure 21:**
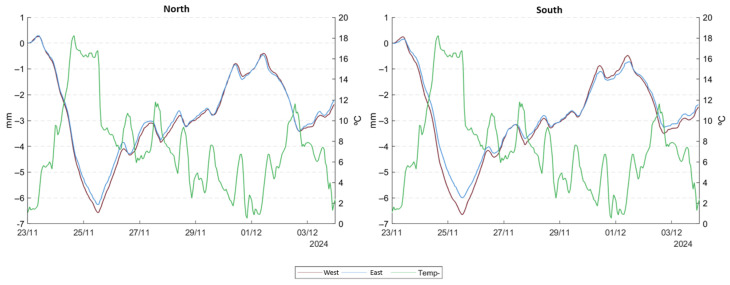
Longitudinal displacement (mm) at the North and South abutments over a 10-day period.

While the inertial data validated the global DAQ performance, the kinematic modules (Node 2) provided direct insights into the local boundary conditions. The measurements captured two distinct kinematic phenomena. Over a multi-day observation window, the longitudinal sensors at the abutments recorded macroscopic drifts directly correlated with the ambient thermal cycles of the girders. As illustrated in Figure 21, the longitudinal displacements at both the North and South abutments were continuously monitored over a 10-day period in late November 2024. The data reveals an inverse correlation with the local temperature, capturing diurnal cycles and broader weather patterns with precision. The total longitudinal movement reached amplitudes of approximately 7 mm. The nearly identical tracking between the East and West sensors at each abutment confirms that the bridge expands and contracts uniformly along its longitudinal axis without significant in-plane rotation or binding.

## 4. Discussion

The results obtained from the Spyckstraße bridge validation campaign demonstrate that the proposed autonomous, offline-synchronized DAQ architecture overcomes the limitations associated with traditional SHM sensor networks. By decoupling data acquisition from the constraints of real-time wireless transmission and extensive cabling, the system provides a highly scalable and reliable solution for capturing high-fidelity structural data.

One of the most persistent challenges in deploying WSNs for SHM is the trade-off between power consumption and data reliability. Continuous radio transmission drastically reduces battery life, while intermittent transmission often leads to packet loss and latency issues in dense civil environments with significant multipath fading [6,8]. The strategy adopted in this study bypassed these wireless bottlenecks. The retrieval of continuous data without dropped frames confirms the robustness of firmware architecture. This methodology proves that complex, energy-draining protocols like PTP or NTP over wireless networks are not strictly necessary when local clocks are sufficiently stable and timestamping is handled at the deterministic hardware interrupt level.

Beyond addressing power and synchronization challenges, achieving high signal integrity was paramount for this system. The high Signal-to-Noise Ratio (SNR) and the identification of resonant peaks in the sub-10 Hz bandwidth (Figure 17) validate that modern, carefully integrated MEMS accelerometers can indeed rival traditional piezoelectric sensors for OMA applications, as suggested by recent literature [14,16]. Similarly, the use of the 24-bit ADS1220 converter in Node 2 allowed string potentiometers to resolve slow thermal drifts.

The spatial resolution achieved through the hybrid strategy provides a comprehensive dataset for accurately mapping the full 3D mode shapes of the Spyckstraße bridge. While the detailed structural analysis and finite element model updating will be explored in a companion paper, the hardware validation presented here establishes the reliability of the baseline data. The ability to simultaneously record global dynamic responses and local kinematic bearing displacements equips structural engineers with a holistic view of the bridge’s health, facilitating more accurate fatigue life estimations and predictive maintenance strategies.

Despite its proven efficacy, the current system design presents certain logistical limitations inherent to offline architectures. The primary constraint is the lack of real-time remote data visibility. Because data is stored locally, system health cannot be monitored from a remote laboratory during the campaign. To handle potential microSD card write errors during long-term deployments, the firmware incorporates a strict byte-count verification step before closing each file. As detailed in Section 2.5, if a write error is detected, a hardware watchdog triggers an automatic system reset to attempt to re-initialize and mount the card. However, if an SD card suffers irreversible hardware corruption or if a battery degrades prematurely, the offline node will silently fail, leading to irrecoverable data loss until the physical retrieval of the equipment.

To mitigate these risks in practical engineering scenarios and ensure long-term service performance without sacrificing the energy benefits of offline storage, future iterations must adopt hybrid Edge-to-Cloud architecture. This involves integrating a low-power, narrow-band communication module. Instead of streaming continuous, power-intensive vibration data over the air, this module would wake up strictly once every 24 h to transmit a minimal diagnostic payload (e.g., remaining battery voltage, internal enclosure temperature, SD card free space, and error flags). If an anomaly is detected, the remote server would immediately alert the maintenance team, drastically minimizing monitoring downtime.

Regarding adaptability to extreme environments, while the DS3231 TCXO provides excellent clock stability across standard industrial ranges, severe thermal gradients in exposed civil structures could still induce cumulative synchronization drifts over multi-month or multi-year deployments. To optimize applicability across various long-term scenarios, the communication module could perform periodic absolute time calibrations against a secure network server. This approach would effectively bound the maximum synchronization error over years of deployment while retaining the ultra-low power consumption and zero-packet-loss reliability of the offline DAQ state machine.

While the field validation proved successful, certain limitations of this study must be acknowledged. First, a no-load laboratory calibration was not performed prior to deployment; the system’s noise floor validation relied on the manufacturer’s specifications and the empirical SNR obtained in situ. Second, although the TCXO limited theoretical clock drift to 0.432 s/day, the specific synchronization errors induced by extreme temperature gradients and battery depletion over long-term deployments were not quantitatively characterized in a controlled environment. Future research should address these metrological characterizations and explore colocated synchronous comparisons with commercial equipment to further benchmark the hardware’s low-level performance.

## 5. Conclusions

This paper presented the design, development, and comprehensive field validation of a fully autonomous, offline-synchronized DAQ architecture specifically tailored for high-density SHM. By shifting away from power-intensive wireless transmission and cumbersome wired setups, the proposed dual-node system reliably leveraged an energy-efficient local-storage paradigm. A robust firmware architecture eliminated the unpredictable write latencies and data loss common in traditional loggers. The implementation of a strict initial UART-based synchronization protocol, paired with high-precision, temperature-compensated RTC to discipline the internal MCU sleep–wake cycles, guaranteed temporal alignment across the network without relying on complex, energy-draining wireless protocols.

The multi-week field deployment on the Spyckstraße bridge demonstrated the system’s operational robustness and scalability. Using a hybrid fixed-roving measurement strategy with 53 independent nodes, the hardware captured both the micro-g global ambient vibrations, yielding clean, high-SNR resonant peaks suitable for OMA, and the sub-millimeter local kinematic movements of the elastomeric bearings under thermal and traffic loads. The proposed architecture offers structural engineers a scalable, energy-efficient, and low-cost solution for monitoring large-scale civil infrastructure, providing the fidelity baseline data essential for advanced structural assessments and digital twin updating.

## Figures and Tables

**Table 1:** Minimum technical design requirements derived from the preliminary experimental campaign.

Subsystem	Parameter	Minimum Requirement
Power Management	Operation mode	Fully Autonomous
	Power source	Standard Alkaline: Type-C
Inertial Measurement	Noise floor	<25 $\;\mu g/\sqrt{\mathrm{Hz}}$
	Measurement axes	Triaxial
Displacement Measurement	Resolution	24-bit ADC
Synchronization	Methodology	Local RTC-based
	Clock stability	±5 ppm (TCXO)
Data Storage	Strategy	Local MicroSD with fast SRAM buffer

**Table 2:** Primary electronic components composing both DAQ nodes.

Subsystem	Component	Manufacturer	Key Specification
Microcontroller (MCU)	STM32L431CCT6	STMicroelectronics	32-bit ARM Cortex-M4 with FPU, ultra-low power, DMA-enabled
Real-Time Clock (RTC)	DS3231MZ	Maxim Integrated	I2C TCXO, ±5 ppm accuracy, 1 Hz PPS output
SRAM Buffer	APS6404L-3SQR-SN	AP Memory	64 MB, QSPI interface, low standby current
MicroSD Interface	Native SDIO/SPI	N/A	High-speed burst-write capable, switched via MOSFET
Power Regulation (LDO)	High-PSRR LDOs	Generic	3.3 V output, ultra-low quiescent current, high rejection ratio
Power Source	3× Type-C Alkaline	Varta	4.5 V nominal, approx. 8000 mAh total capacity

**Table 3:** Technical specification comparison of high-end industrial MEMS accelerometers.

Characteristic	ADXL355	SCA3300-D01	IIS3DWB
Focus	Structural Health Monitoring (SHM), inclinometry, IoT	High-precision inclinometry, heavy machinery	High-frequency vibration analysis
ADC Resolution	20 bit	16 bit	16 bit
Measurement Range	±2 g, ±8 g	±1.5 g, ±3 g	±2 g, ±4 g, ±8 g, ±16 g
Noise Density	25 $\mu g/\sqrt{\mathrm{Hz}}$	37 $\mu g/\sqrt{\mathrm{Hz}}$	45 $\mu g/\sqrt{\mathrm{Hz}}$
Temp. Drift	0.15 mg/°C	0.15 mg/°C	0.3 mg/°C
Bandwidth	Up to 1 kHz	10 Hz to 70 Hz	Up to 6.3 kHz
Current Consumption	200 µA	1.2 mA	1.1 mA
Digital Interface	SPI/I2C	SPI	SPI
Temperature Range	−40 °C to +125 °C	−40 °C to +125 °C	−40 °C to +105 °C

**Table 4:** Technical specification comparison of Analog-to-Digital Converters (ADCs).

Characteristic	ADS1220	AD7124-4	STM32L431 Internal ADC
Architecture	Sigma-Delta	Sigma-Delta	SAR
Resolution	24-bit	24-bit	12-bit (16-bit with oversampling)
Max. Speed (SPS)	2 kSPS	19.2 kSPS	5 MSPS
Gain (PGA)	Yes, 1 to 128	Yes, 1 to 128	No true integrated PGA
Differential Inputs	2 differential/4 single-ended	4 differential/7 single-ended	Single-ended/Limited differential
Typical Consumption	120 µA (duty-cycle mode)	255 µA (low-power mode)	200 µA/MSPS (dynamic)
Excitation Sources	10 µA to 1.5 mA	Multiple options	No
50/60 Hz Line Filter	Simultaneous at 20 SPS	Simultaneous at 25 SPS	No
Data Interface	SPI	SPI/QSPI/DSP	Internal MCU Bus

**Table 5:** Vibration modes resulting from the calibration and validation process of the Digital Twin of the Spyckstraße Bridge.

Section	Mode	Mode Description	Measured Freq. (Hz)	Calibrated Model Freq. (Hz)	Error
North Span	1	First-order vertical bending	3.75	3.79	0.97%
	2	First-order torsion around the longitudinal axis	4.16	4.09	−1.64%
	3	Second-order torsion around the longitudinal axis	5.41	5.57	2.96%
Central Span	1	First-order vertical bending	3.58	3.60	0.45%
	2	First-order torsion around the longitudinal axis	3.82	3.85	0.83%
	3	Transverse bending + first-order torsion	4.88	4.90	0.46%
	4	Second-order torsion around the longitudinal axis	5.60	5.47	−2.32%
South Span	1	First-order vertical bending	3.85	3.90	1.21%
	2	First-order torsion around the longitudinal axis	4.29	4.23	−1.43%
	3	Second-order torsion around the longitudinal axis	5.74	5.69	−0.89%
North Pier	1	Longitudinal bending of the north pier	1.96	1.98	1.13%
	2	Transverse bending of the north pier	2.14	2.16	0.99%
South Pier	1	Longitudinal bending of the south pier	1.98	1.98	0.11%
	2	Transverse bending of the south pier	2.17	2.16	−0.41%

## Data Availability

The raw binary data and custom processing scripts generated during the validation campaign are available from the corresponding author upon reasonable request.

## Abbreviations

The following abbreviations are used in this manuscript:

ADC	Analog-to-Digital Converter
DAQ	Data Acquisition
DMA	Direct Memory Access
DOF	Degree of Freedom
EMI	Electromagnetic Interference
EXTI	External Interrupt
FDD	Frequency Domain Decomposition
FFT	Fast Fourier Transform
FPU	Floating-Point Unit
I2C	Inter-Integrated Circuit
IEPE	Integrated Electronics Piezo-Electric
IoT	Internet of Things
LDO	Low-Dropout Regulator
MCU	Microcontroller Unit
MEMS	Micro-Electro-Mechanical Systems
NB-IoT	Narrowband Internet of Things
NTP	Network Time Protocol
ODR	Output Data Rate
OMA	Operational Modal Analysis
PCB	Printed Circuit Board
PGA	Programmable Gain Amplifier
PSD	Power Spectral Density
PSRAM	Pseudo-Static Random-Access Memory
PSRR	Power Supply Rejection Ratio
PTP	Precision Time Protocol
QSPI	Quad Serial Peripheral Interface
RTC	Real-Time Clock
SD	Secure Digital
SHM	Structural Health Monitoring
SNR	Signal-to-Noise Ratio
SPI	Serial Peripheral Interface
SVD	Singular Value Decomposition
TCXO	Temperature-Compensated Crystal Oscillator
WSN	Wireless Sensor Network
